# Improved Quantification of Cell Density in the Arterial Wall—A Novel Nucleus Splitting Approach Applied to 3D Two-Photon Laser-Scanning Microscopy

**DOI:** 10.3389/fphys.2021.814434

**Published:** 2022-01-12

**Authors:** Koen W. F. van der Laan, Koen D. Reesink, Myrthe M. van der Bruggen, Armand M. G. Jaminon, Leon J. Schurgers, Remco T. A. Megens, Wouter Huberts, Tammo Delhaas, Bart Spronck

**Affiliations:** ^1^Department of Biomedical Engineering, CARIM School for Cardiovascular Diseases, Maastricht University, Maastricht, Netherlands; ^2^Department of Biochemistry, CARIM School for Cardiovascular Diseases, Maastricht University, Maastricht, Netherlands; ^3^Institute for Cardiovascular Prevention, Ludwig Maximilian University, Munich, Germany; ^4^German Center for Cardiovascular Research (DZHK), Partner Site Munich Heart Alliance, Munich, Germany; ^5^Department of Biomedical Engineering, School of Engineering and Applied Science, Yale University, New Haven, CT, United States

**Keywords:** cell content characterization, image analysis, nucleus segmentation, spectral clustering, cell density distribution, vascular smooth muscle cell apoptosis

## Abstract

Accurate information on vascular smooth muscle cell (VSMC) content, orientation, and distribution in blood vessels is indispensable to increase understanding of arterial remodeling and to improve modeling of vascular biomechanics. We have previously proposed an analysis method to automatically characterize VSMC orientation and transmural distribution in murine carotid arteries under well-controlled biomechanical conditions. However, coincident nuclei, erroneously detected as one large nucleus, were excluded from the analysis, hampering accurate VSMC content characterization and distorting transmural distributions. In the present study, therefore, we aim to (1) improve the previous method by adding a “nucleus splitting” procedure to split coinciding nuclei, (2) evaluate the accuracy of this novel method, and (3) test this method in a mouse model of VSMC apoptosis. After euthanasia, carotid arteries from SM22α-hDTR *Apoe*^–/–^ and control *Apoe*^–/–^ mice were bluntly dissected, excised, mounted in a biaxial biomechanical tester and brought to *in vivo* axial stretch and a pressure of 100 mmHg. Nuclei and elastin fibers were then stained using Syto-41 and Eosin-Y, respectively, and imaged using 3D two-photon laser scanning microscopy. Nuclei were segmented from images and coincident nuclei were split. The nucleus splitting procedure determines the likelihood that voxel pairs within coincident nuclei belong to the same nucleus and utilizes these likelihoods to identify individual nuclei using spectral clustering. Manual nucleus counts were used as a reference to assess the performance of our splitting procedure. Before and after splitting, automatic nucleus counts differed −26.6 ± 9.90% (*p* < 0.001) and −1.44 ± 7.05% (*p* = 0.467) from the manual reference, respectively. Whereas the slope of the relative difference between the manual and automated counts as a function of the manual count was significantly negative before splitting (*p* = 0.008), this slope became insignificant after splitting (*p* = 0.653). Smooth muscle apoptosis led to a 33.7% decrease in VSMC density (*p* = 0.008). Nucleus splitting improves the accuracy of automated cell content quantification in murine carotid arteries and overcomes the progressively worsening problem of coincident nuclei with increasing cell content in vessels. The presented image analysis framework provides a robust tool to quantify cell content, orientation, shape, and distribution in vessels to inform experimental and advanced computational studies on vascular structure and function.

## Introduction

Vascular smooth muscle cells (VSMCs) are a crucial component of blood vessels because they regulate extracellular matrix (ECM) production, maintain mechanical homeostasis in larger vessels, and enable autoregulation in smaller vessels (Owens, 1995; Brozovich et al., 2016). Changes in VSMC functionality, phenotype, and content, potentially lead to changes in diameter, wall thickness, structure, and mechanical properties of the vessel (Jaminon et al., 2019). Accurate information about a vessel’s VSMC content, orientation, and distribution under well-controlled conditions is indispensable to improve modeling of active biomechanics of the vessel wall, thereby helping to further our understanding of the role VSMC play in vascular remodeling.

VSMC content is commonly determined from histological cross-sections of vessels stained with a nucleic acid dye (Clarke et al., 2010; Pai et al., 2011; Yu et al., 2011; Roostalu et al., 2018). VSMC density and distribution estimates obtained from histological cross sections are potentially disturbed, as this histological technique requires fixation and sectioning of the vessel. In addition, experience shows that it is difficult to control the orientation of smaller vessels fixed in paraffin wax for sectioning, making it nearly impossible to obtain accurate VSMC orientation data from histological cross sections of small vessels. Thus, while histology is a useful tool for assessing VSMC content of vessels, it seems ill-suited for providing accurate, quantitative information about VSMC density and distributions, especially in murine vessels.

To address this problem, we previously developed a method that uses two-photon laser scanning microscopy (TPLSM) to image intact pressurized and stretched mouse carotid arteries stained with a nucleic acid fluorescent dye (Spronck et al., 2016). Using this method, one can quantify the transmural location and/or orientation distributions of VSMC nuclei under well-controlled mechanical conditions in 3D, avoiding the need to fixate and section the vessel. Our results showed that VSMCs in mouse carotid arteries have significant non-zero helix angles and distinctly layered transmural distributions. This level of detail contrasts with computational models of artery biomechanics, in which VSMCs are often assumed to be oriented exclusively circumferentially (Zulliger et al., 2004; Masson et al., 2011; Spronck et al., 2015).

A significant limitation of our previous method for quantifying VSMC nucleus content is that nuclei which are situated closely together may be identified as a single nucleus (Spronck et al., 2016). Consequently, the shape and orientation of the resulting identified structures do not resemble those of normal nuclei and, if noticed, these structures may/should be excluded from further analysis. In our previous study, on average 6.5% of the detected nuclear structures had to be rejected because they exceeded the maximum size threshold. Furthermore, it is possible for structures within the size thresholds to consist of multiple nuclei. Clearly, correct identification of nucleus structures would enable more accurate estimates of VSMC density and distribution.

While several methods exist for splitting segmented structures consisting of multiple nuclei into their corresponding nuclei, these are generally optimized for spherical nuclei, or for image stacks with consistent contrast levels throughout, or developed for 2D images (Danìk et al., 2009; Mathew et al., 2015; Atta-Fosu et al., 2016; Abdolhoseini et al., 2019; Ruszczycki et al., 2019). VSMC nuclei are typically twice to six times as long as they are wide and applying the above-mentioned methods to multiple elongated nuclei structures often leads to over segmentation (Daly et al., 2002). Additionally, non-fixated tissue cannot be optically cleared, resulting in decreasing contrast with increasing sample penetration. Although contrast compensation methods exist for TPLSM image stacks, these will be difficult to apply due to the curved and multilayered nature of vessels. Hence, existing methods are ill-suited for splitting multiple elongated nuclei segmented from TPLSM image stacks of intact, non-fixated vessels.

The aim of the present paper is to develop a procedure for splitting structures of touching elongated nuclei and to evaluate the accuracy of the resulting automatic characterization of the blood vessel’s cell density. The performance of the “nucleus splitting” procedure will be compared with manually determined nucleus counts from TPLSM data obtained from mouse carotid arteries containing a wide range of cell density.

## Materials and Methods

### Experimental Animals and Vessel Samples

All animal studies were performed under an approved protocol by the Ethics Committee for animal experiments of Maastricht University. In the present study, we utilized an inducible VSMC apoptosis mouse model (*SM22α-hDTR Apoe^–/–^*, Clarke et al., 2006). A total of 12 *Apoe^–/–^* animals were used, six of which had the *hDTR* knock-in receptor (*SM22α-hDTR Apoe^–/–^*) and served as the VSMC apoptosis group, while the other six (control *Apoe^–/–^*) served as the control group. After 8 weeks, both groups were treated with diphtheria toxin (DT), inducing VSMC apoptosis in the *hDTR* knock-in receptor group but not in the control group. Both groups were euthanized through an overdose of isoflurane 2 weeks after the injection. Left carotid arteries, generally 5–7 mm in length, were harvested from the animals. After isolation, as much connective tissue as possible was bluntly removed from the arteries. Samples were immediately stored at 4 degrees Celsius in Hank’s balanced salt solution (HBSS) (Thermo Fisher Scientific, Paisley, United Kingdom) containing 1.0 μM sodium nitroprusside (Sigma-Aldrich, St. Louis, MO, United States) to ensure full vasodilation.

### Sample Mounting and Preparation

**FIGURE 1:**
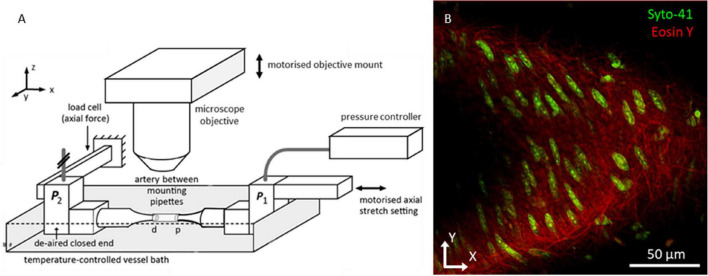
Biomechanical testing setup allowing for imaging vessels under in *vivo*-like conditions. **(A)** Overview bi-axial biomechanical testing set-up. Samples were mounted with both ends fixed at glass pipettes. Pressure and axial force were measured at the closed-end distal pipet (*P*_2_). While measuring the axial force, the motorized proximal pipet was used to bring artery to its *in vivo*-like length. The setup was placed under the microscope with the objective situated above the artery. The motorized objective mount facilitated scanning through the sample along the *z*-axis to create a z-stack. **(B)** Representative two photon fluorescence microscopy image of mouse carotid artery wall. Green channel shows cell nuclei; red channel shows the elastin fiber network. Because vessels did not always lay flat in the imaging plane, the vessel wall tended to take on a parabolic shape in the imaging plane, as visible in this example.

Within 4 h after excision, samples were mounted on glass pipets in a custom biaxial biomechanical testing setup (van der Bruggen et al., 2021), a schematic of which is shown in Figure 1A. Samples were stretched to their *in vivo-*like length and pressurized to 100 mmHg, as described previously (van der Bruggen et al., 2021). Finally, 1.5 μM Syto-41 (Thermo Fisher Scientific, Paisley, United Kingdom) and 0.5 μM Eosin-Y (Sigma-Aldrich, St. Louis, MO, United States) were added to the organ bath to stain nuclei and elastin fibers, respectively. Samples were left to stain for 30 min before imaging. Syto-41 and Eosin-Y were left in the organ bath during imaging.

### Imaging Setup

Fluorescence microscopy images were acquired using a Radiance2100 two-photon laser scanning setup (Bio-Rad, Hercules, CA, United States) equipped with a 60x CFI APO NIR Objective (NA 1.0, WD 2.8 mm) (Nikon, Minato City, Tokyo, Japan). The center wavelength of the Tsunami tunable pulsed femtosecond laser (Spectra-Physics, Santa Clara, California, United States) was tuned to 810 nm. Using preinstalled optical filters in the microscope, the spectral ranges of two detection channels were set to 440–510 and 580–650 nm, and displayed in green and red, respectively. In this configuration, the green channel captured the light emitted by the Syto-41 staining, whilst the red channel captured the light emitted by the Eosin-Y staining, the results of which can be seen in Figure 1B. These ranges were chosen to minimize overlapping fluorescence emission spectra within the channels. Image resolution was set to 1,024 × 1,024 pixels^2^ with a pixel size of 0.2 × 0.2 μm^2^, resulting in a field of view of 205 × 205 μm^2^. Microscope focus was positioned above the vessel and images were taken sequentially, moving down 0.45 μm between every image, until the near vessel wall was passed completely. In this manner, image stacks were made for every sample, generally 30–40 μm deep.

### Image Stack Analysis

**FIGURE 2:**
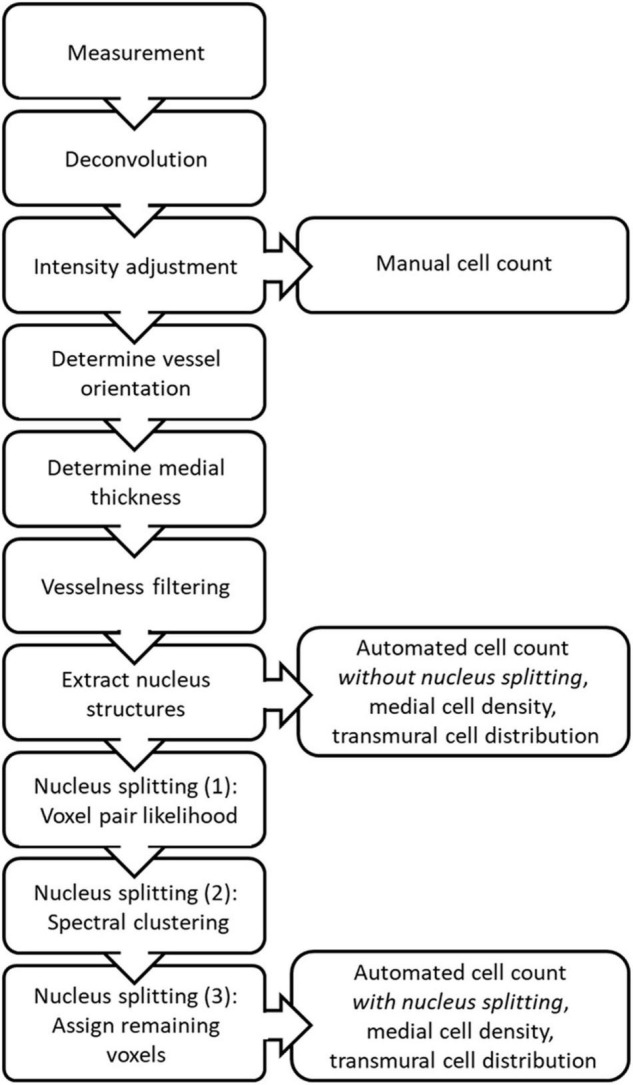
Schematic overview of the steps taken during image stack analysis to produce the manual and automated counts, medial cell densities and transmural cell distributions, with and without nucleus splitting.

The steps in the proposed image stack analysis method are displayed in Figure 2. The analysis method produces manual and automated counts, without and with splitting, as well as medial cell densities and transmural cell density distributions derived from the automated counts.

### Deconvolution

Recorded image stacks were sharpened using Huygens professional image deconvolution software (Scientific Volume Imaging, Hilversum, Netherlands). The classic maximum likelihood estimation algorithm was used to perform the deconvolution. For each channel of the image stack, a theoretical point spread function was constructed from the microscopic parameters mentioned previously (microscope type, excitation and emission wavelengths, embedding medium refractive index, voxel dimensions, objective magnification, and numerical aperture) and entered into the program. The deconvolution algorithm was set to run for a maximum of thirty iterations or until a quality threshold, set to 0.005, was reached. All further image analysis for nucleus quantification was performed using custom scripts written in MATLAB R2019b (MathWorks, Natick, MA, United States).

### Determination of Vessel Orientation

**FIGURE 3:**
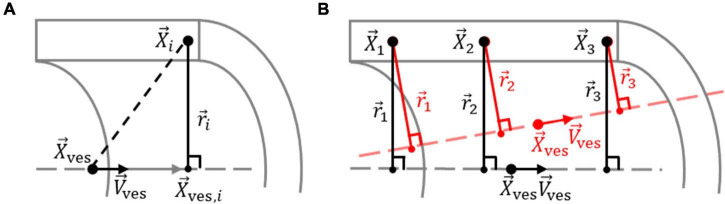
Determination of vessel centerline from elastin voxels. **(A)** Definition of the vector ${\vec{r}}_{i}$ between elastin voxel *i* in the vessel wall, at location ${\vec{X}}_{i}$, and the point along the vessel centerline, defined by orientation unit vector ${\vec{V}}_{\text{ves}}$ through location ${\vec{X}}_{\text{ves}}$, closest to voxel *i*, at location ${\vec{X}}_{\text{ves},i}$. **(B)** The optimum vessel centerline is determined by optimizing ${\vec{X}}_{\text{ves}}$ and ${\vec{V}}_{\text{ves}}$ so that the variation in the length of ${\vec{r}}_{i}$ for the *n* elastin voxels is minimized. An optimized and non-optimized centerline for the elastin voxels 1, 2, and 3 are shown in black and red, respectively.

A straight cylinder was fitted through the obtained elastin data to determine the vessel centerline. For this procedure, voxels from the red (elastin) channel were used when they exceeded a background threshold of 15% of the maximum possible intensity value. The optimal straight cylinder was determined by finding the centerline position and orientation for which the variance in the radial distance from the voxels to the centerline was minimized. The radial distance from a voxel to the vessel centerline equals the minimal distance from the centerline to that voxel and is determined by the length of the vector, ${\vec{r}}_{i},$ between the voxel and the point along the centerline closest to the voxel, as defined by $r_{i}=\left|{\vec{r}}_{i}\right|={\left(r_{i,1}^{2}+r_{i,2}^{2}+r_{i,3}^{2}\right)}^{1/2}$. The radial distance was calculated according to


(1) $$r_{i}=\left|{\vec{X}}_{i}-{\vec{X}}_{\text{ves},i}\right|,$$

where ${\vec{X}}_{i}$ describes the position vector of voxel *i* and ${\vec{X}}_{\text{ves},i}$ describes the position vector of the point along the vessel centerline closest to ${\vec{X}}_{i}$. The vessel centerline is defined by ${\vec{X}}_{\text{ves}}$ and ${\vec{V}}_{\text{ves}}$, the position vector of a point along the centerline and the orientation unit vector of the centerline, respectively. ${\vec{X}}_{\text{ves},i}$ is found by moving the magnitude of the projection of $\left({\vec{X}}_{i}-{\vec{X}}_{\text{ves}}\right)$ on the centerline in the direction of ${\vec{V}}_{\text{ves}}$ from ${\vec{X}}_{\text{ves}}$, as shown in Figure 3A, according to


(2) $${\vec{X}}_{\text{ves},i}={\vec{X}}_{\text{ves}}+{\vec{V}}_{\text{ves}}\left({\vec{V}}_{\text{ves}}\cdot \left({\vec{X}}_{i}-{\vec{X}}_{\text{ves}}\right)\right).$$

The vessel centerline was determined by optimizing ${\vec{X}}_{\text{ves}}$ and ${\vec{V}}_{\text{ves}}$ so that the variance in *r_i_* was minimized for the *n* selected elastin voxels, as shown in Figure 3B, using the cost (error) function


(3) $$\varepsilon_{\text{ves}}\left(r_{i}\right)=\sum_{i=1}^{n}\frac{{\left(r_{i}-\bar{r}\right)}^{2}}{n-1},$$

where $\bar{r}$ represents the expected value of *r_i_* for the *n* selected elastin voxels. The cost function was minimized by varying the first and third coordinates of ${\vec{X}}_{\text{ves}}$ and ${\vec{V}}_{\text{ves}}$ using a trust-region-reflective non-linear least-squares algorithm. The second coordinate of ${\vec{X}}_{\text{ves}}$ was kept fixed at half the width of an image stack to ensure that the least squares algorithm would converge to a single point along the vessel centerline for ${\vec{X}}_{\text{ves}}$.

### Medial Thickness Determination

**FIGURE 4:**
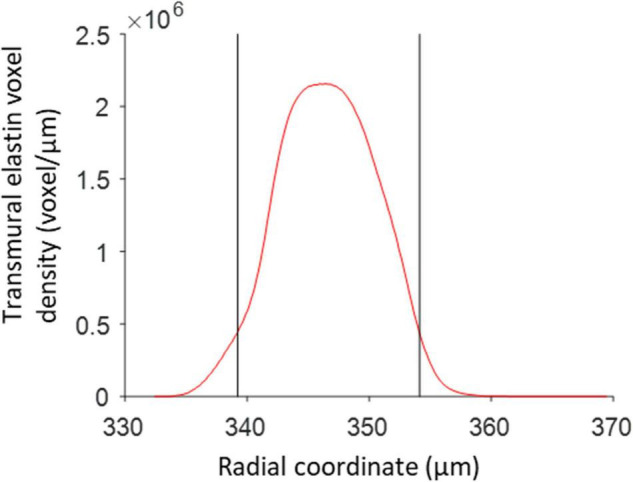
Media borders determined from the transmural elastin voxel density distribution. The two black vertical lines correspond to the radii, with respect to the vessel centerline, where the elastin density first and last exceeds 20% of the maximum elastin density. These locations are used as the radial borders of the vessel media.

The radial positions of the inner and outer borders of the media, with respect to the vessel centerline, were determined using voxels in the red channel that exceeded a background threshold of 6% of the maximum possible intensity value. A transmural density distribution of these voxels was determined using the *ksdensity* MATLAB function. The distribution was evaluated for the complete range of radial distances corresponding with the selected voxels, with a resolution of 0.1 μm and with a bandwidth of 0.8 for the kernel smoothing window (Bowman and Azzalini, 2004). The borders of the media were set at the edges of the region where the density distribution exceeds 20% of the maximum value of the distribution (Figure 4).

### Manual Nucleus Counting

The number of nuclei within each 3D image were counted by two independent experienced people (KWFL and RTAM), and the average count per scan was used as a reference to determine the accuracy of the image analysis method.

### Vesselness Filtering

After deconvolution, the intensity of the green channel was adjusted so that the lowest 99.6% of voxels, based on intensity, spanned the whole intensity range. The previously reported Vesselness filtering-based approach was adopted to enhance elongated structures in the green channel and suppress background noise (Spronck et al., 2016). 3D kernels based on the 2nd order Gaussian derivatives with a standard deviation of 1.2 μm in all three directions were used. Kernel voxel size was chosen to fit ± 3 standard deviations in each direction, yielding a kernel size of 37 × 37 × 17 voxels. Vesselness filtering parameters α, β, and *c* were all set to 0.15 and an intensity threshold of 0.1 was subsequently used to segment the nuclei from the Vesselness-filtered image stacks into binary image stacks. These parameters were chosen to visually give the best trade-offs between sensitivity and reliability in enhancing nuclei, as well as minimize the frequency of multiple enhanced nuclei touching each other.

In addition to enhancing elongated structures, Vesselness filtering was used to assign each voxel the orientation of the elongated structure at that location. These orientations correspond to the smallest absolute eigenvalue produced by the Hessian matrix used during Vesselness filtering. The orientation of voxels in an elongated nucleus were therefore predominantly in line with the orientation of that nucleus.

### Nucleus Extraction

Nucleus structures were extracted from binary image stacks using a 3D 6-connectivity neighborhood connected-component analysis. This groups high binary voxels that are directly in front, behind, above, below, left, or right of each other into structures representing nuclei. A minimum volume threshold, set to 54 μm^3^, was applied to the extracted structures to exclude structures that were too small to reasonably be nuclei. The number of remaining nucleus structures was used as the automated cell count, without nucleus splitting.

### Transmural Cell-Density Distribution Estimation

Radial locations of the *n* extracted nucleus structures compared to the vessel centerline, *r*_c,*i*_, were determined from their centroids using


(4) $$r_{\text{c},i}=\left|{\vec{X}}_{\text{c},i}-\left({\vec{X}}_{\text{ves}}+{\vec{V}}_{\text{ves}}\left({\vec{V}}_{\text{ves}}\cdot \left({\vec{X}}_{\text{c},i}-{\vec{X}}_{\text{ves}}\right)\right)\right)\right|,$$

where ${\vec{X}}_{\text{c},i}$ represents the structure centroid location for extracted nucleus structure *i*. A transmural density distribution of *r*_c,*i*_ was determined using the *ksdensity* MATLAB function. The distribution was evaluated for the complete range of radial distances corresponding with the selected structures, using a resolution of 0.1 μm and with a bandwidth of 1 for the kernel smoothing window (Bowman and Azzalini, 2004).

### Nucleus Splitting

The proposed nucleus splitting procedure was used to detect whether extracted nucleus structures consisted of multiple nuclei and, if so, to split these structures into their separate nuclei. The procedure consists of three major steps: (1) determining the likelihood for each combination of two voxels within a structure that they are part of the same nucleus based on their location and orientation; (2) using these likelihoods to build a Laplacian matrix for spectral clustering and identify individual nucleus cores; and (3) assigning voxels that were excluded from the Laplacian matrix to cores according to their highest likelihood of belonging to a specific core. While the last step is not strictly necessary for improving cell count accuracy, it does allow further analysis of split nuclei in terms of size, shape, or orientation analysis.

#### Step 1: Voxel Pair Likelihood Determination

The likelihood that any two voxels in a structure are part of the same nucleus was determined based on the assumptions that VSMC nuclei are predominantly long and cylindrically-shaped, organized in distinct layers within the vessel wall, and oriented primarily in the circumferential-axial plane, compared to the vessel centerline. A likelihood that two voxels are part of the same nucleus was defined for each of these assumptions.

The first likelihood, *p*_rd,*i*,*j*_ ∈ [0, 1], is determined using the shortest distance from voxel *i* to the line represented by the location and orientation of voxel *j*, *r*_*i*,*j*_, and the shortest distance from voxel *j* to the line represented by the location and orientation of voxel *i*, *r*_*j*,*i*_. Since the orientation of a voxel is in line with the elongated structure at that location, if both *r*_*i*,*j*_ and *r*_*j,i*_ are relatively short, compared to the thickness of the elongated structure, the voxels are likely part of the same elongated structure. The likelihood *p*_rd,*i,j*_ was calculated according to Gaussian distribution


**FIGURE 5:**
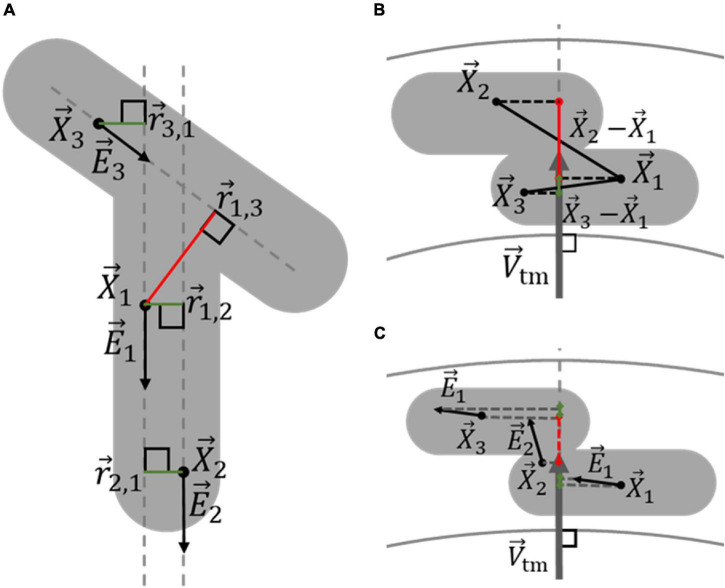
Determination of the likelihood that two voxels belong to the same nucleus, based on their orientation and location. **(A)** Voxels within a multiple-nucleus structure, shown in gray, can still likely be part of the same elongated structure, and thus the same nucleus, even when relatively far apart. Voxels 1 and 2, with locations ${\vec{X}}_{1}$ and ${\vec{X}}_{2}$, respectively, have similar orientations, given by ${\vec{E}}_{1}$ and ${\vec{E}}_{2}$, respectively. Because the line between ${\vec{X}}_{1}$ and ${\vec{X}}_{2}$ is relatively in line with both ${\vec{E}}_{1}$ and ${\vec{E}}_{2}$, vectors ${\vec{r}}_{1,2}$ and ${\vec{r}}_{2,1}$ are both short enough to produce high likelihoods that voxels 1 and 2 belong to the same nucleus, indicated by the green lines. On the other hand, because the orientation of voxel 3, ${\vec{E}}_{3}$, is dissimilar to that of the line between ${\vec{X}}_{1}$ and ${\vec{X}}_{3},{\vec{r}}_{1,3}$ is too long for voxel 1 and 3 to likely be part of the same nucleus, indicated by the red color, even though ${\vec{r}}_{3,1}$ is small. **(B)** The transmural distance between voxels influences whether they are likely part of the same VSMC layer, and thus the same nucleus. The projection of ${\vec{X}}_{1}$-${\vec{X}}_{3}$ on ${\vec{\text{V}}}_{\text{tm}}$ is relatively small, making it likely that voxel 1 and 3 belong to the same VSMC layer. Conversely, the projection of ${\vec{X}}_{1}$-${\vec{X}}_{2}$ on ${\vec{\text{V}}}_{\text{tm}}$ is relatively large, making it unlikely that voxel 1 and 2 belong to the same VSMC layer. **(C)** The transmural components of voxel orientations indicate whether voxels are oriented in the axial-circumferential plane of the vessel, similar to VSMCs, and whether they likely belong to a specific nucleus. The green and red colors of the projections of ${\vec{E}}_{1}$, ${\vec{E}}_{2},$ and ${\vec{E}}_{3}$ on ${\vec{\text{V}}}_{\text{tm}}$ indicates voxels 1 and 3 are oriented primarily in the axial-circumferential plane, while voxel 2 is not.

(5) prd,i,j=e(-ri,j2-rj,i22σr2),

with the standard deviation σ_r_ set to 0.8 μm, based on the width of a VSMC nucleus (O’Connell et al., 2008). Similar to the radial location of nucleus centroids compared to the vessel centerline, *r*_*i,j*_ and *r*_*j,i*_ were calculated according to


(6) $$r_{i,j}=\left|{\vec{X}}_{i}-\left({\vec{X}}_{j}+{\vec{E}}_{j}\left({\vec{E}}_{j}\cdot \left({\vec{X}}_{i}-{\vec{X}}_{j}\right)\right)\right)\right|,$$

and


(7) $$r_{j,i}=\left|{\vec{X}}_{j}-\left({\vec{X}}_{i}+{\vec{E}}_{i}\left({\vec{E}}_{i}\cdot \left({\vec{X}}_{j}-{\vec{X}}_{i}\right)\right)\right)\right|,$$

where ${\vec{X}}_{i}$ and ${\vec{X}}_{j}$ are the position vectors of voxels *i* and *j*, respectively, and ${\vec{E}}_{i}$ and ${\vec{E}}_{j}$ denote the orientations of voxel *i* and *j*, respectively. *p*_*rd,i,j*_ is defined so that it will be high if the voxels are situated close together, regardless of their orientation, as *r*_*i,j*_ and *r*_*j,i*_ will always be equal to or smaller than the distance between the two voxels. However, the value of *p*_rd,*i*,*j*_ can still be high when the voxels are relatively far away if their positions and orientations are all in line, as illustrated in Figure 5A. This allows for voxel pairs situated at opposite ends of the same elongated nucleus to have a high *p*_rd,*i*,*j*_.

The second likelihood, *p*_td,*i*,*j*_ ∈ [0, 1], was determined using the transmural distance between voxels *i* and *j*, *d*_td,*i,j*_. If this distance is relatively large, compared to the thickness of a nucleus, then the two voxels likely lay in separate VSMC layers and, as illustrated in Figure 5B, were therefore unlikely to be part of the same nucleus. The likelihood *p*_td,*i,j*_ was calculated according to the Gaussian distribution


(8) ptd,i,j=e-dtd,i,j22σtd2,

with the standard deviation σ_td_ set to 0.8 μm, based on the thickness of a VSMC nucleus in the vessel media. The transmural distance between voxels *i* and *j*, *d*_td,*i,j*_, was determined by the magnitude of the positioning vector between the voxels *i* and *j* on the transmural orientation unit vector at the centroid of the structure to be split according to


(9) $$d_{td,i,j}=\frac{{\vec{V}}_{\text{tm}}\cdot \left({\vec{X}}_{i}-{\vec{X}}_{j}\right)}{\left|{\vec{V}}_{\text{tm}}\right|}.$$

Here ${\vec{V}}_{\text{tm}}$ represents the transmural orientation vector and was defined by the shortest possible vector from the vessel centerline to the structure’s centroid according to


(10) $${\vec{V}}_{\text{tm}}={\vec{X}}_{\text{c}}-\left({\vec{X}}_{\text{ves}}+{\vec{V}}_{\text{ves}}\left({\vec{V}}_{\text{ves}}\cdot \left({\vec{X}}_{\text{c}}-{\vec{X}}_{\text{ves}}\right)\right)\right).$$

Here *X*_c_ is the location of the centroid of the structure that is to be split, given by the average location of the voxels comprising the structure.

The third likelihood, *p*_ta,*i*,*j*_ ∈ [0, 1], was determined by whether the orientation of voxels *i* and *j* were primarily in the circumferential-axial plane of the vessel. Voxels in between touching nuclei from different layers typically had a transmural orientation and, because VSMCs are oriented in the circumferential-axial plane, it was difficult to discern which nucleus these voxels belonged to. Rather than informing whether two voxels are likely part of the same nucleus, *p*_ta,*i,j*_ serves as an exclusion criterion of transmurally orientated voxels for determining nucleus cores in the next nucleus splitting step. By excluding transmural oriented voxels that do not clearly belong to a specific nucleus, the reliability and accuracy of spectral clustering for determining nucleus cores was improved. *p*_ta,*i,j*_ is calculated using the magnitude of the voxel orientations unit vectors ${\vec{E}}_{i}$ and ${\vec{E}}_{j}$ on the transmural orientation vector, ${\vec{V}}_{\text{tm}}$, according to


(11) $$p_{\text{ta},i,j}=\left(1-\frac{\left|\left({\vec{V}}_{\text{tm}}\cdot {\vec{E}}_{i}\right)\right|}{\left|{\vec{V}}_{\text{tm}}\right|}\right)\left(1-\frac{\left|\left({\vec{V}}_{\text{tm}}\cdot {\vec{E}}_{j}\right)\right|}{\left|{\vec{V}}_{\text{tm}}\right|}\right).$$

In this manner, *p*_ta,*i,j*_ only produces a high likelihood if both voxel orientations are primarily along the circumferential-axial plane of the vessel, as illustrated in Figure 5C.

#### Step 2: Nucleus Core Determination

Spectral clustering uses the similarity between the data points in a dataset to define clusters of similar data points (von Luxburg, 2007). In the nucleus splitting procedure a normalized spectral clustering algorithm was adopted to cluster structure voxels into cores representing the separate nuclei by using the likelihood that voxels were part of the same nucleus as the similarity between them. The adopted algorithm requires the construction of a normalized random walk Laplacian matrix from the weighted adjacency matrix **R** and the degree matrix **D** according to


(12) $${\text{L}}_{\text{rw}}=\text{I}-{\text{D}}^{-1}\text{R},$$

where **I** is the identity matrix. The weighted adjacency matrix **R,** is a symmetric matrix in which the columns and rows represent the voxels in the structure and the non-diagonal elements describe the undirected similarity between voxels. The elements of the weighted adjacency matrix were calculated by multiplying the three likelihoods that two voxels within a structure were part of the same nucleus with each other according to


(13) $$\text{R}=\left[\rho_{i,j}\right]=\left\{ \begin{array}{c} \begin{array}{cc} p_{\text{rd},i,j}\cdot p_{\text{ta},i,j}\cdot p_{\text{td},i,j}, & i\neq j\\ 0, & i=j \end{array} \end{array}\right.,$$

in which ρ_*i*,*j*_ ∈ [0, 1] denotes the similarity between voxels *i* and *j* are part of the same nucleus. The degree matrix **D** contains along its diagonal the degree of each voxel, *d_i_*, which describes the combined similarity of that voxel with all voxels in the structure and is otherwise filled with zeros. The degree of a voxel is determined by its summed similarity with all voxel in the structure according to


(14) $$d_{i}=\sum_{j=1}^{n}\rho_{i,j}.$$

During spectral clustering, a degree threshold was chosen below which voxels were excluded from the Laplacian matrix. This improved the accuracy and reliability of the algorithm as visually it was confirmed that, without the exclusion of low degree voxels, the algorithm inconsistently produced additional cores in and between nuclei. On the other hand, if the threshold was set too high, it was possible that small nuclei were not assigned to a core as most voxels in that core were excluded. The degree threshold was set at *d*_*min*_ = 40 as this visually produced the most consistent results.

The spectral clustering algorithm utilizes the eigenvalues and eigenvectors of the Laplacian matrix to cluster structure voxels that exceeded the degree threshold into nucleus cores. The algorithm was limited to split a structure in no more than *k* = 10 cores. Consequently, the *k* smallest absolute eigenvalues, with their corresponding eigenvectors, were determined from the Laplacian matrix using a Krylov method. First, it was determined whether a structure consisted of a single or multiple nuclei. If the total volume of voxels exceeding the degree threshold was smaller than 1.8 μm^3^, the structure was deemed to consist of a single nucleus as there were not enough voxels to facilitate multiple cores. Additionally, if the first and second eigenvalue were smaller than 0.01 and larger than 0.02, respectively, indicating that all voxels are sufficiently likely to be part of the same nucleus with each other, the structure was deemed to consist of a single nucleus.

If the structure was not deemed to consist of a single nucleus, the algorithm performed *k*-means clustering using the eigenvectors of the Laplacian matrix corresponding to the *k* smallest absolute eigenvalues as cluster indicators to create nucleus cores (Bowman and Azzalini, 2004). The highest average silhouette value, describing how close a voxel is to its own core centroid compared to other core centroids, was used to determine the *k* cores to split the structure in, with *k* ranging from 2 to 10 (Rousseeuw, 1987). To prevent the *k*-means clustering algorithm from converging to a local minimum due to a poor starting point, it was repeated 20 times for each value of *k*. The result with the smallest mean within-cluster point-to-centroid distance was selected for each value of *k*, as this value is small for well-grouped clusters.

#### Step 3: Assignment of Low-Degree Voxels to Cores

In the last step of the nucleus splitting procedure, the voxels which were excluded from the Laplacian matrix, were divided between the nucleus cores to produce whole nuclei. Voxels were allocated to the core which they were deemed to have the highest likelihood of belonging to. This likelihood was based on the shortest distance from a voxel to a core centerline and the distance from a voxel to the edge of a core in the direction of the core centroid. The likelihood *p*_*ij*_ ∈ [0, 1] that voxel *i* belonged to core *j*, was calculated according to


**FIGURE 6:**
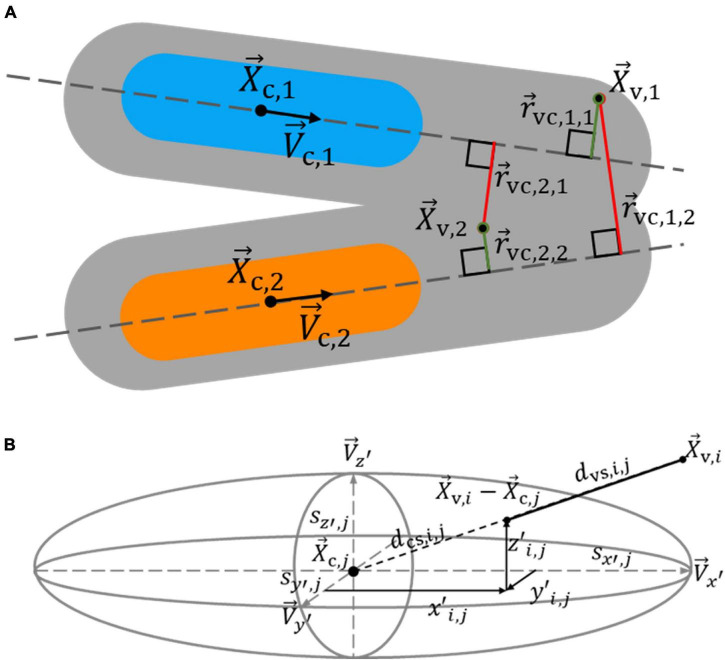
Determination of the likelihood that voxels excluded from spectral clustering belong to a nucleus core. An excluded voxel is assigned to a nucleus core based on its distance to core centerlines and the distance to core surfaces, as estimated by an ellipsoid. **(A)** The shortest distance from excluded voxels 1 and 2 to core centerlines 1 and 2 influences which core they likely belong to. Since ${\vec{r}}_{\mathrm{vc},1,1}$ is shorter than ${\vec{r}}_{\mathrm{vc},1,2}$, indicated by the green and red colors, respectively, voxel 1 is more likely to belong to core 1 than 2, shown in blue and orange, respectively. Voxel 2 more likely belongs to core 2 than 1, as ${\vec{r}}_{\mathrm{vc},2,2}$ is shorter than ${\vec{r}}_{\mathrm{vc},2,1}$. **(B)** The graph shows the path from excluded voxel *i* to the surface of core *j*, as estimated by an ellipsoid, in the direction of the core centroid. A voxel is more likely to belong to a core it has a shorter distance to its surface to.

(15) pi,j=e(-rvc,i,j22σvc2)e(-dvs,i,j22σvs2),

where *r*_vc,*i,j*_ and *d*_vs,*i,j*_ are the shortest distance from voxel *i* to core centerline *j* and the distance from voxel *i* to the surface of core *j*, in the direction of core centroid *j*, respectively. Standard deviations σ_*vc*_ and σ_*vs*_ were set to 2 and 5 μm, respectively. The vector *r*_vc,*i,j*_ was calculated according to


(16) $$r_{\text{vc},i,j}=\left|{\vec{X}}_{\text{v},i}-\left({\vec{X}}_{\text{c},j}+{\vec{V}}_{\text{c},j}\left({\vec{V}}_{\text{c},j}\cdot \left({\vec{X}}_{\text{v},i}-{\vec{X}}_{\text{c},j}\right)\right)\right)\right|,$$

where ${\vec{X}}_{\text{c},j}$ and ${\vec{X}}_{\text{v},i}$ represent core centroid *j* and excluded voxel *i* locations, respectively, and ${\vec{\text{V}}}_{\text{c},j}$ represents the core centerline *j* orientation vector. Figure 6A illustrates how ${\vec{r}}_{\text{vc},i,j}$ was used to assign excluded voxels to cores.

Core centerline orientations were determined in one of two ways. If a core consisted of 12 voxels or less, the core centerline orientation was calculated from the mean orientation of the voxels in the core, and then normalized. Otherwise, the core centerline orientation was determined by finding the orientation on which the summed projected magnitude of core voxel orientations was maximized by minimizing the cost (error) function


(17) $$\varepsilon_{\text{co}}\left({\vec{V}}_{\text{c},j}\right)=\sum_{i=1}^{n}\left(1-\left|\left({\vec{E}}_{\text{c},i}\cdot {\vec{V}}_{\text{c},j}\right)\right|\right),$$

using a trust-region-reflective non-linear least-squares algorithm. Here ${\vec{E}}_{\text{c},i}$ represent the orientations of the core’s voxels.

To determine the distance from an excluded voxel to the edge of a core, an ellipsoid approximation of the core’s shape was made. The eigenvectors of the covariance matrix of the core’s voxel locations were used as the principal axes of the ellipsoid, while two times the square root of the eigenvalues of the covariance matrix were used as the size of the ellipsoid along those axes (Nielsen and Bhatia, 2014). The distance from excluded voxel *i* to the surface of the ellipsoid approximation of core *j* in the direction of the core’s centroid, *d*_*vs*_*_,i,j_*, was determined, as illustrated in Figure 6B, according to


(18) $$d_{\text{vs},i,j}=\left|{\vec{X}}_{\text{v},i}-{\vec{X}}_{\text{c},j}\right|-d_{\text{cs},i,j}.$$

Here ${\vec{X}}_{\text{v},i}$ and ${\vec{X}}_{\text{c},j}$ are the position vectors of excluded voxel *i* and the centroid of core *j*, and *d*_cs,*i,j*_ represents the distance from the centroid to the surface of core *j* in the direction of ${\vec{X}}_{\text{v},i}$. The distance *d*_ce,*i,j*_ was calculated from the length along each of the principle ellipsoid axes at which the vector ${\vec{X}}_{\text{v},i}$$-{\vec{X}}_{\text{c},j}$crosses the ellipsoid surface, defined as x′i,j, y′i,j, and z′i,j, according to


(19) dcs,i,j=x′i,j2+y′i,j2+z′i,j2.

The distances x′i,j, y′i,j, and z′i,j, were calculated by multiplying the size of a core ellipsoid along each axis, *s*_*x’,j*_, *s*_*y’,j*_, and *s*_*z’,j*_, with the amplitude of the projection of the normalized vector between ${\vec{X}}_{\text{c},j}$ and ${\vec{X}}_{\text{v},i}$ on the corresponding axis, ${\vec{V}}_{x^{\prime }}$, ${\vec{V}}_{y^{\prime }},$ and ${\vec{V}}_{z^{\prime }}$, respectively, according to


(20) x′i,j=sx′,j(V→x′,j⋅(X→c,j-X→v,i)|X→c,j-X→v,i|),

(21) y′i,j=sy′,j(V→y′,j⋅(X→c,j-X→v,i)|X→c,j-X→v,i|),

and


(22) z′i,j=sz′,j(V→z′,j⋅(X→c,j-X→v,i)|X→c,j-X→v,i|).

### Determination of Medial Cell Density

The media volume within an image stack was determined by multiplying the number of voxels that had a radial distance to the vessel centerline between the inner and outer borders of the media with the volume of one voxel (18⋅10^–3^ μm^3^). The nucleus count, with identical radial distance restrictions, was divided by the media volume to determine the media cell density for the sample.

### Choice of Image Analysis Parameters

Table 1 lists all image analysis parameter values used in this study. These values where chosen based on preliminary results, using manual nucleus counts as a reference. Parameter estimation was limited to six of the 12 scans: three from both the control and VSMC apoptosis groups, whilst the image analysis procedure’s performance was judged using all 12 scans. In this manner we sought to evaluate whether the proposed image analysis method improved nucleus structure identification, instead of only determining which parameters provided the best results for this dataset.

**TABLE 1:** Parameter values for nucleus quantification from TPLSM 3D images.

Parameter description	Value	Unit	Step in protocol
Red channel background threshold	15	%*	Vessel orientation

Red channel background threshold	6	%*	Medial thickness determination
Kernel smoothing function bandwidth	1.2	−	
Transmural distribution threshold for edge detection	20	%**	

Second-order Gaussian kernel SD	1.2	μm	Vesselness filtering
Vesselness filtering parameter α	0.15	−	
Vesselness filtering parameter β	0.15	−	
Vesselness filtering parameter *c*	0.15	−	
Intensity threshold for nucleus detection	0.1	−	

Min. size nucleus structure	54	μm^3^	Nucleus extraction

Kernel smoothing function bandwidth	1.0		Cell density distribution estimation

Voxel-voxel radial distance SD (σ_r_)	0.8	μm^3^	Nucleus splitting, step 1
Transmural distance SD (σ_td_)	0.8	μm^3^	

Minimum degree for spectral clustering (*d*_min_)	40	−	Nucleus splitting, step 2
Min. structure size for splitting threshold	1.8	μm^3^	
Max. spectral clustering 1st eigenvalue	0.01	−	
Min. spectral clustering 2nd eigenvalue	0.02	−	
Number of *k*-means clustering repetitions	20	−	
Max. number of cores (k)	10	−	

Voxel-core radial distance SD (σ_vc_)	2.0	μm	Nucleus splitting, step 3
Voxel-ellipsoid surface distance SD (σ_vs_)	5.0	μm	
Voxel count threshold for core centerline determination	12	Voxels	

*Percentage with respect to maximum possible channel intensity value; **percentage with respect to maximum transmural distribution value. SD, standard deviation.

### Statistical Analysis

Paired *t*-tests were used to determine whether differences between the mean absolute manual and automated nucleus counts were significant. One-sample two-sided *t*-tests were used to determine whether relative differences between manual nucleus count and automated nucleus counts were significant. Linear regression was used to analyze the relation of the relative differences between the automated and manual nucleus counts with the manual nucleus count. Lastly, independent *t*-test was used to analyze whether the media cell density of the VSMC apoptosis group was significantly different from the control group, using the automated nucleus count with the nuclear splitting procedure. All statistical tests were performed using two tails and with α = 0.05.

## Results

### Nucleus Count Without Splitting

**FIGURE 7:**
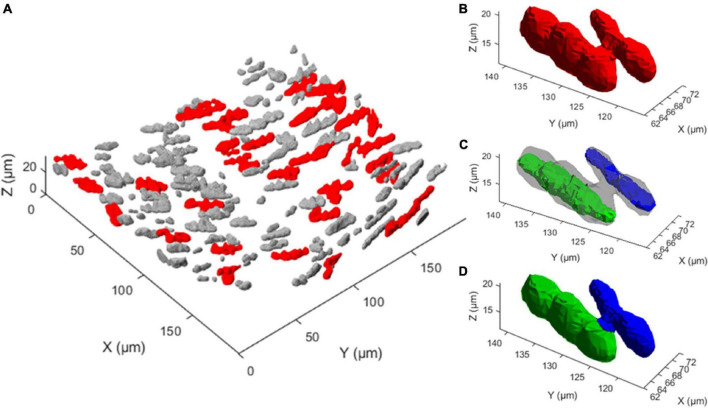
Multi-nucleus structure splitting is required for accurate nucleus quantification. **(A)** 3D rendering of the extracted nucleus structures from one 3D fluorescence image. Gray structures were deemed a single nucleus by the nuclear splitting procedure, while red structures were deemed to consist of multiple nuclei, illustrating how frequently multiple nucleus structures appear. **(B)** A single extracted structure, shown in red, that visually consists of two touching nuclei. **(C)** Two nucleus cores, shown in green and blue, are the result of the first two steps of the nuclear spitting procedure on the structure shown in **(B)**. **(D)** Result of the nuclear splitting procedure, where the structure in panel B has been separated into two structures, indicated in green and blue.

**FIGURE 8:**
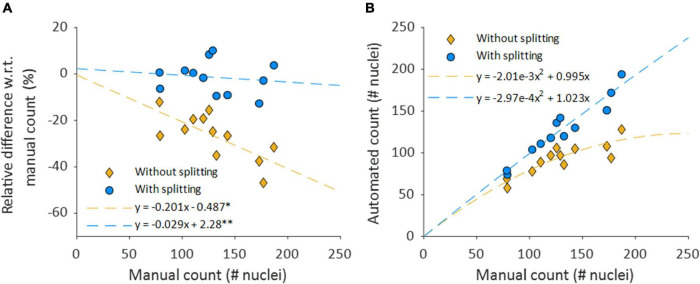
Nuclear splitting improves accuracy of cell content quantification. **(A)** Shows the differences between the manual and automated counts, normalized with respect to the manual count. The dashed lines depict simple linear regression lines of both counts. *p = 0.008 and *p* = 0.953 for the slope and intercept, respectively. **p = 0.653 and *p* = 0.789 for the slope and intercept, respectively. **(B)** Automated nucleus counts without and with splitting, against the manual count. The formulas for the dashed lines in panel B are directly derived from the linear regression coefficients of their respective relative data shown in **(A)**.

Mean automated nucleus counts without nucleus splitting are significantly smaller than manual counts (Δ = −36.9, *p* < 0.001) (Table 2). Mean relative differences between manual and automated nucleus counts without splitting were significant as well (Δ = −26.6% *p* = <0.001). Visually, it was confirmed that this mismatch was primarily caused by closely situated nuclei that were recognized as a single structure, as is demonstrated in Figure 7A. Linear regression showed that the relative underestimation of the number on nuclei by the automated count without splitting increased significantly with increasing sample cell density (slope = −0.201, *p* = 0.008) (Figure 8). This indicates that the error from identifying multiple nuclei as a single structure got progressively worse with increasing cell density.

**TABLE 2:** Nuclear splitting results in insignificant differences between mean manual and automated nucleus counts.

Method/Measure	Unit	*n*	Mean ± SD	*p*-value
Manual reference			129.8 ± 35.7	–
Automated count without splitting	Nuclei per scan	12	92.9 ± 18.8	< 0.001*
Automated count with splitting			127.6 ± 34.9	0.467*

Relative difference manual and automated count without splitting	%	12	−26.6 ± 9.90	< 0.001**
Relative difference manual and automated count with splitting			−1.44 ± 7.05	0.493**

Control group	10^4^ nuclei per mm^3^	6	10.9 ± 2.57	–
VSMC apoptosis group			7.23 ± 0.85	0.008***

Percentages for the relative differences are calculated with respect to manual count results. * Paired t-test vs. manual count. ** One sample t-test. *** Two sample t-test vs. control group. SD, standard deviation.; n, number of image stacks included in analysis.

### Nucleus Count With Nucleus Splitting

The accuracy of the automated nucleus count improved when splitting structures of multiple elongated nuclei with the nucleus splitting procedure, as the mean manual and automated counts was no longer significantly different (Δ = −2.2, *p* = 0.467) (Table 2). Furthermore, mean relative differences between manual and automated nucleus counts with splitting were no longer significant (Δ = −1.44% *p* = 0.493). Linear regression shows that the difference between manual and automated count with splitting no longer significantly depends on the number of nuclei in the sample (slope = −0.029, *p* = 0.653) (Figure 8A).

### Transmural Nucleus Distribution

**FIGURE 9:**
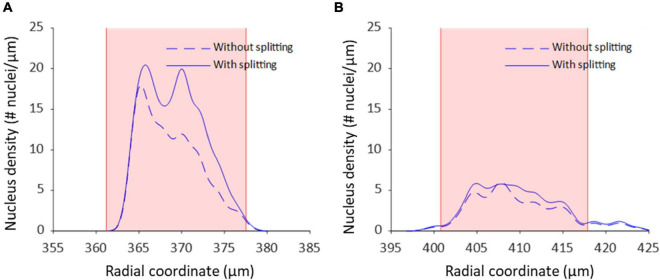
Nucleus splitting makes VSMC layers more distinct in transmural cell density distributions. **(A)** Nucleus density as a function of radial location for a sample of a wild type animal (control *Apoe^–/–^*) with high cell content. By splitting structures with nuclei from different layers the layered organization of VSMCs becomes more prominent. Multi-nucleus structures are predominantly on the adventitial side of the vessel wall. **(B)** Nucleus density as a function radial location for a sample from the VSMC apoptosis group (*SM22α-hDTR Apoe^–/–^*) with low cell content. Multi-nucleus structures are less frequent and spread more evenly throughout the vessel wall of the low cell content vessel compared to the high cell content vessel. Shaded (pink) areas denote the media in each sample.

Nucleus splitting has a marked impact on the transmural nucleus density distributions produced from the automated counts (Figure 9). Since the procedure increased the number of nuclei counted in each sample, the area beneath the transmural nucleus distribution curves increased correspondingly. Additionally, the local maxima were more pronounced in the transmural cell distribution curves of high cell content samples (Figure 9A). Interestingly, multiple nuclei were more frequently extracted as a single structure near the adventitial side of the vessel wall compared to the luminal side.

### Induced Vascular Smooth Muscle Cell Apoptosis Mouse Model

The medial nucleus density of the VSMC apoptosis group was, on average, 33.7% lower than the control group in the induced VSMC apoptosis case study (Table 2). Besides the decrease in cell content, fewer strongly pronounced local maxima and minima were present in the transmural nucleus distributions for the VSMC apoptosis group (Figure 9).

## Discussion

The aim of this study was to develop a method for splitting touching elongated nucleus structures segmented from TPLSM image stacks of intact non-fixated blood vessels into their corresponding nuclei and to evaluate the accuracy of the automatic characterization of the blood vessel’s cell content.

The results show that the spectral clustering-based nucleus splitting procedure successfully split structures of multiple elongated nuclei into their individual nuclei, as illustrated in Figure 7, based on the likelihood that voxels within those structures were part of the same elongated nucleus. The implementation of the nucleus splitting method significantly improved the accuracy of the automated nucleus count (Table 2). In addition to this, the relative difference between the manual and automated counts was no longer significantly dependent on the samples cell content (Figure 8). With regard to transmural nucleus distributions, the nucleus splitting procedure generally made local maxima more pronounced, making the layered distribution of VSMCs in the media more apparent (Figure 9). This effect was stronger in vessels with higher cell density, because touching nuclei from different layers needed to be split more often. This makes the nucleus splitting procedure valuable, especially for higher cell density vessels, as these have more nuclei situated close enough to be recognized as a single structure.

While previous studies have analyzed VSMCs segmented from fluorescence microscopy image stacks of non-sectioned vessels before, their focus was on pattern and orientation analysis, rather than cell content quantification (Daly et al., 2002; McGrath et al., 2005; Spronck et al., 2016; Cordoba and Daly, 2019). So far, histological tissue analysis has been the standard for quantifying cell content in vessels (Clarke et al., 2010; Pai et al., 2011; Yu et al., 2011; Roostalu et al., 2018). The proposed image analysis method provides a useful new tool for quantifying cell content in vessels from 3D image stacks. Compared to histology, our method provides several benefits as it does not require vessel fixation and sectioning, making it possible to determine cell content, densities and distributions within the vessel wall under *in vivo*-like loading conditions without disturbing them. Therefore, our method can help provide potential new insights and possibilities for research on active vessel biomechanics, vessel pathophysiology, and modeling of the vessel wall.

The induced VSMC apoptosis case study served to illustrate the usefulness of the proposed image analysis method as the measured 33.7% decrease in medial nucleus density for the VSMC apoptosis group, as compared to the control group, was in line with literature (Clarke et al., 2006). Additionally, the transmural distributions of VSMC apoptosis group displayed less profound local maxima compared to the control group, as illustrated in Figure 9, indicating that induced VSMC apoptosis disturbed the layered distribution of VSMCs in the vessel wall. This level of detailed transmural cell distribution analysis would not be feasible with histological slides, as vessel cell content would not be quantified under well-controlled biomechanical *in vivo*-like conditions.

Interestingly, throughout the dataset multi-nucleus structures were more frequent at the adventitial than the luminal side of the vessel wall, as demonstrated in Figure 9. It is likely that, as fluorescence intensity decreases with increased sample penetration, dim extra-nuclear fluorescent structures became undetectable and no longer linked neighboring nuclei. Alternatively, it could be that spacing between nuclei increased toward the luminal side of the vessel wall, resulting in fewer multi-nucleus structures. Perhaps the intravenous injection of DT, and subsequent penetration into the vessel wall from the luminal side, may have caused an uneven level of cell death throughout the vessel, thereby influencing nucleus spacing. However, the current dataset is insufficient to determine whether this observation was due to pathophysiological model properties or imaging artifacts.

While the proposed nucleus splitting procedure significantly improved the accuracy of automated cell content quantification, the spread in the relative differences between automated and manual counts remained similar (Table 2). Because the Syto-41 fluorescent staining also labeled mRNA to a lesser extent, other structures were visible near nuclei in the green channel throughout the dataset, to various extents. Consequently, parameters α, β, *c*, and the intensity threshold used during the Vesselness filtering step were tuned to filter out (most of) these structures, preventing them from acting as bridges between extracted nuclei and resulting in more multi-nucleus structures. Conversely, this makes the Vesselness filtering less sensitive to low intensity nuclei, and more sensitive to brightness variations throughout and between samples, resulting in a larger spread in relative differences the automated and manual counts. To minimize this problem, it is advisable for future research to use a more selectively nucleic acid stain.

The memory required to perform the proposed image stack analysis method should be considered when selecting a computer to run the image stack analysis on. Since large Laplacian matrixes are generated when splitting large multi-nucleus structures, it is possible to run out of memory when processing these structures. While a modern computer, with an i7-9700 CPU @ 3.00 GHz (Intel, Santa Clara, CA, United States) and 16 GB of RAM, was used to process the data, some image stacks had to be excluded from the dataset as they contained excessively large structures that could therefore not be processed. To prevent the exclusion of image stacks, for future research, it is recommended to use a computer with more memory or further optimize the code for large structures.

## Conclusion

The proposed nucleus splitting procedure greatly improves the accuracy of the automated quantification of cell content in mouse carotid arteries. The presented image analysis framework now provides a robust tool to quantitatively characterize VSMC content, orientation and distribution to inform experimental and advanced computational studies on vascular structure and function.

## Data Availability Statement

The raw data supporting the conclusions of this article will be made available by the authors, without undue reservation.

## Ethics Statement

The animal study was reviewed and approved by the Ethics Committee for Animal Experiments of Maastricht University.

## Author Contributions

KWFL: conceptual design, setting up imaging protocol and data analysis procedure, performing manual nucleus count and data analysis, and drafting the article. KDR and BS: conceptual design, data interpretation, and critical revision of the article. MMB: sample preparation and data collection. AMGJ and LJS: provided animal model. RM: contributed to setting up imaging protocol and performing manual nucleus count. WH: critical revision of the article, with a focus on the mathematical aspects of the image analysis method. TD: critical revision of the article. All authors contributed to the article and approved the submitted version.

## Conflict of Interest

The authors declare that the research was conducted in the absence of any commercial or financial relationships that could be construed as a potential conflict of interest.

## Publisher’s Note

All claims expressed in this article are solely those of the authors and do not necessarily represent those of their affiliated organizations, or those of the publisher, the editors and the reviewers. Any product that may be evaluated in this article, or claim that may be made by its manufacturer, is not guaranteed or endorsed by the publisher.
